# Primary mesenteric hydatid cyst; a rare manifestation of hydatid disease through a case report and literature review

**DOI:** 10.1016/j.ijscr.2022.107592

**Published:** 2022-09-05

**Authors:** Mohammad Asef Adelyar, Samea Hesham, Khalida Walizada, Ershad Ahmad Mushkani, Ramin Saadaat

**Affiliations:** a Department of Abdominal Surgery, Aliabad Teaching Hospital, Kabul University of Medical Sciences, Kabul, Afghanistan; b Department of Neurosurgery, Aliabad Teaching Hospital, Kabul University of Medical Sciences, Kabul, Afghanistan; c Department of Pharmacology, Kabul University of Medical Sciences, Kabul, Afghanistan; d Department of Histopathology, French Medical Institute for Children, Kabul, Afghanistan

**Keywords:** Hydatid cyst, Mesentery, Echinococcosis granulosus, Hydatidosis

## Abstract

**Introduction and importance:**

Hydatidosis, a common zoonotic disease, especially in countries which are poorly developed, is not only capable of affecting a huge number of humans but also animals.

**Case presentation:**

A 40-year-old female presented to our hospital complaining of the left flank and left upper quadrant pain increasing with exertion for three years. Physical findings revealed a firm lump in the left upper quadrant area extending to the left flank about (15cmx14.5 cm) in size. Abdominal CT scan reported a well defined low attenuating fluid density consistent with thick walled cystic lesion in the left upper quadrant, which is not separable from the lower pole of left kidney as well as pancreatic tail, showing significant mass effect over the left renal pelvis resulting in moderate dilatation of left pelvi-calyceal system superiorly. Our case was managed with the removal of laminated membrane of the cyst and followed by Mebendazole therapy.

**Clinical discussion:**

The disease is commonly caused by Echinococcosis granulosus, the parasite mostly takes place in the liver (70 %) and in the lungs (25 %). In 13 % of cases the primary hepatic cyst raptures causing the formation of intraperitoneal cyst, however the primary intraperitoneal hydatid cyst (2 %) and the primary mesenteric hydatid cysts are very rare. Considering the possibility of hydatid cyst especially in the endemic regions is highly recommended because in these regions there are many diversities in the presentation. As surgical excision with subsequent Mebendazole therapy for four months is the proper treatment for these cases.

**Conclusion:**

Primary hydatid cyst of the mesentery is rare even in the endemic regions. Thus, it's important to differentiate hydatid disease of abdomen from the other cystic lesions, occured in the abdominal cavity, specially in the endemic regions.

## Highlights

• Primary hydatid cyst of mesentery is rare even in the endemic regions
• Surgery is the treatment of choice
• Peritoneal cystic mass must be consider in differential diagnosis.

## 1 Introduction

The disease is commonly caused by Echinococcosis granulosus, this parasite mostly takes place in the liver (70 %) and in the lungs (25 %). In 13 % of cases the primary hepatic cyst raptures causing the formation of intraperitoneal hydatid cyst, however the primary intraperitoneal hydatid cyst (2 %) and the primary mesenteric hydatid cysts are very rare. In this article, the author presents a case of primary mesenteric hydatid cyst with chronic pain in left flank and left upper quadrant of abdomen [1]. Our work has been reported in concordance with the SCARE criteria [2].

## 2 Case report

**Fig. 1:**
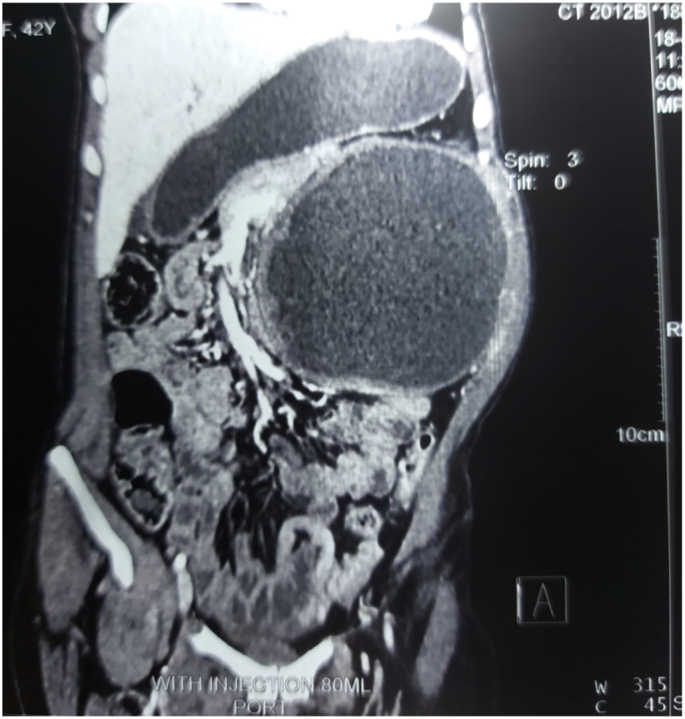
Coronal section of abdominal CT scan showing low attenuating well defined in the left upper quadrant of abdomen with mild hydronephrosis left pelvi-calyceal system.

**Fig. 2:**
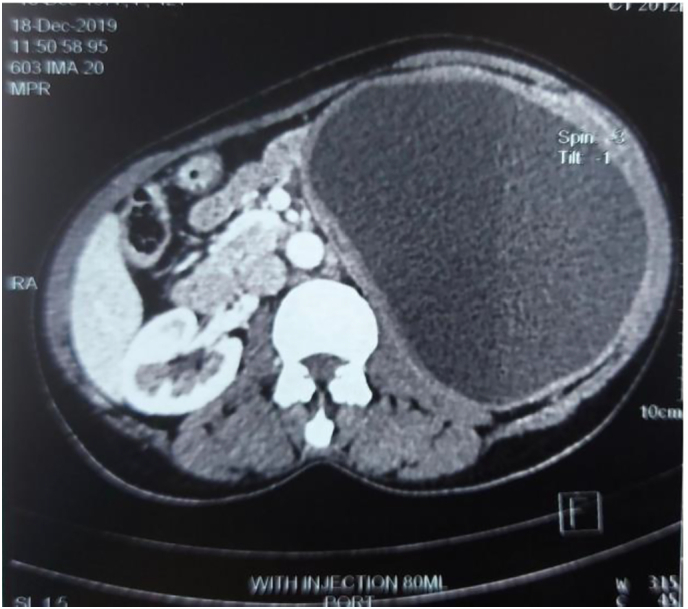
This horizontal section of abdominal CT scan indicates a well-defined cystic mass in the left side of the abdomen.

**Fig. 3:**
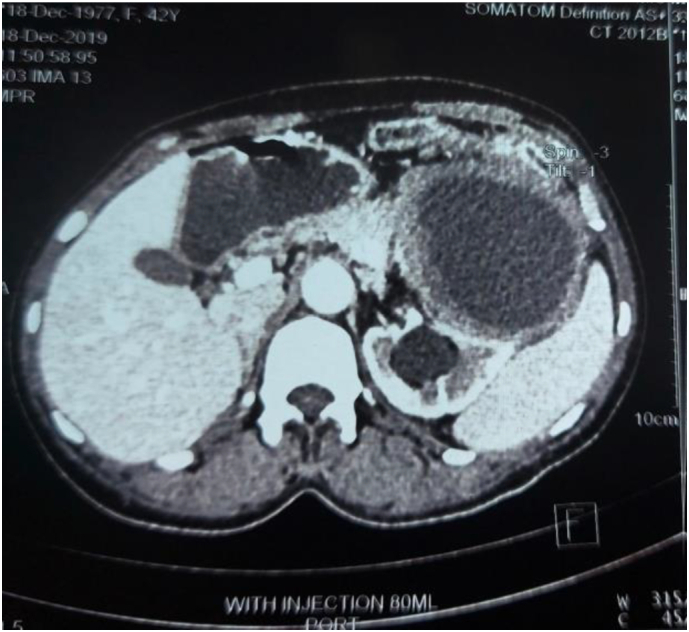
Transverse section of abdominal CT scan indicating a thick-walled, well-defined, hypodense, cystic lesion attached to the tail of pancreas.

**Fig. 4:**
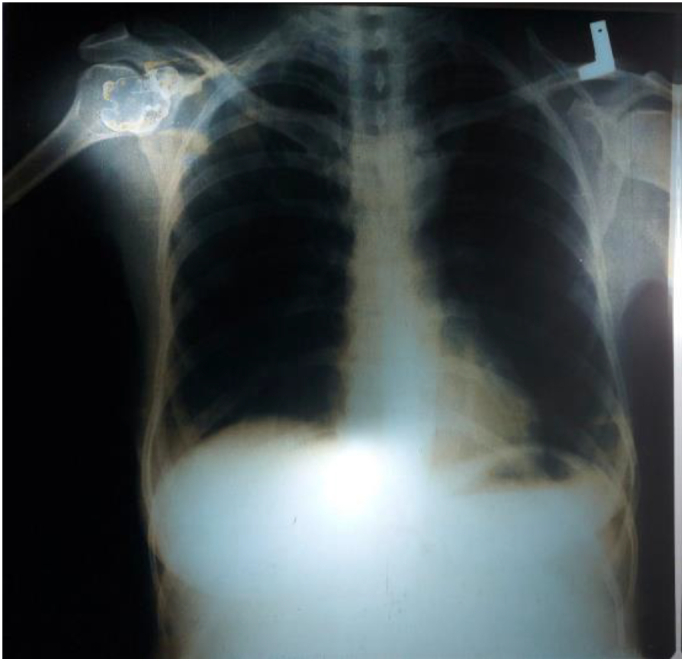
Showing, Normal chest radiograph.

**Fig. 5:**
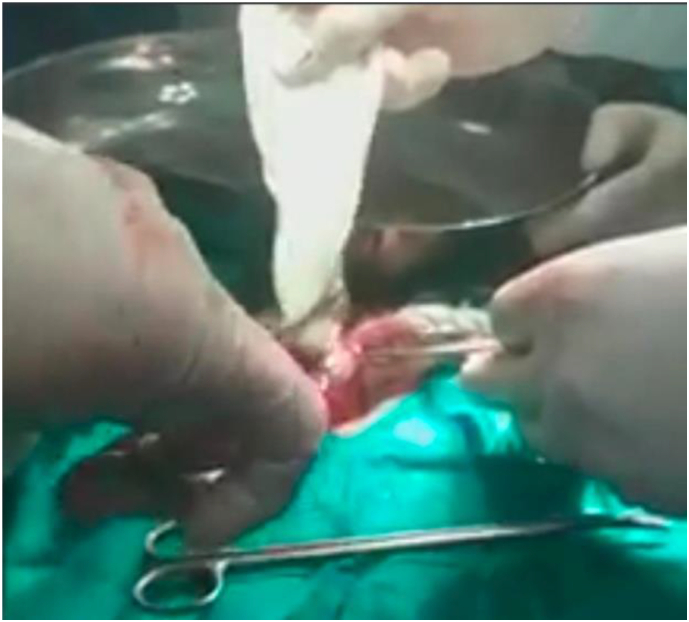
Intra-operative image, showing Removal of Laminated Membrane of the cyst.

**Fig. 6:**
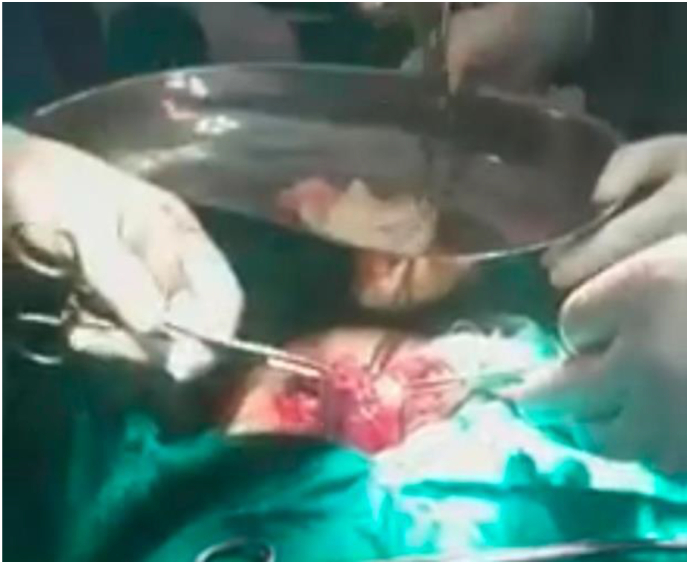
Intra-operative image, showing the cyst was completely removed.

A 40-year-old female presented to the hospital complaining of the left flank and left upper quadrant pain increasing with exertion since three years. The patient is married, with no remarkable past medical, allergic, drug and medication history. Physical findings consist of a firm lump in the left upper quadrant area extending in the left flank with the size of (15 cm × 14.5 cm). The mass was mildly tender on palpation and dull on percussion as well. Blood exam results turned out with HB11g%, TLC-8000, Differential Leukocyte count- P64, L30, E5, M1, and B0. Liver and Kidney function tests were normal. Ultrasonography demonstrated a well-defined large cystic lesion in the left upper quadrant of abdomen which had close attachment with inferior pole of the left kidney; minimal hydronephrosis with hydroureter were also observed during ultrasonography. It was not determined whether the mass was neoplastic or other abdominal cystic mass. Liver, Pancreas, Spleen and other organs were found normal. Intravenous Urography has revealed large cyst involving lower pole of the left kidney associated with displacement of collecting system and mild hydronephrosis. Abdominal CT scan reported well defined low attenuating fluid density thick walled cystic lesion in the left upper quadrant which is not separable from the lower pole of left kidney as well as pancreatic tail and the lesion shows significant mass effect over left renal pelvis resulting in moderate dilatation of left pelvi-calyceal system superiorly. The first imaging hypothesis could be infected hydatid cyst; considering the mesenteric cyst the differential diagnosis (Fig. 1, Fig. 2, Fig. 3, Fig. 4, Fig. 5, Fig. 6).

We prepared the patient for Laparotomy with Albendazole twice daily for 1 month. The operation was performed in Ali-Abad Teaching Hospital. On exploration a cystic mass with the size of (14.5 cm × 15 cm × 15 cm) in the mesentery jejunum strictly adhesive with the adjacent organ; liver, pancreas, spleen and other organs were normal. After instillation of sodium chloride hypertonic 20 % inside cyst it was completely excised. However, due to low economic resource, we could not able to perform the histopathologic and serological tests.

The operation was successful and the patient was advised to take Albendazole twice daily for one month. During three years of follow-up with multiple visits at Ali-Abad Teaching Hospital the patient didn't complain of any symptoms.

## 3 Discussion

The larvae of the tapeworm Echinococcosis infest as hydatid disease. Humans are mostly infected by Echinococcosis Granulosus species. The Sheep farming and Cattle farming areas such as Mediterranean Sea, Asia North and East Africa, South America, Australia and the Middle East are the regions in which most cases of this parasite are found [3]. Two hosts are inhabited by parasites during its lifecycle: a definitive host (usually a dog) and an intermediate host such as humans [3], [4].

There are two ways of acquiring this parasite by humans such as direct contact by infected dog and ingestion of the infected food. Embryos reach the liver in 60 %–70 % cases when they migrate through intestinal mucosa and traveling through intestinal lymphatics and venules. The embryos can be carried by blood stream to any organ if they bypass the liver [5]. These embryos mostly reside in the liver (59–75 %), followed by the lungs (27 %), kidneys (3 %), bones (1–4 %) and the brain (1–2 %). Other parts of the body which are rarely infected are the heart, spleen, pancreas, omentum, ovaries, parametrium, pelvis, thyroid, orbit or retroperitonem and muscles [3], [5].

Even though a few primary cases of peritoneal hydatid cyst have been detected, almost all of the peritoneal hydatid cysts are secondary to hepatic cysts. 13 % of abdominal hydatid cyst resides in the peritoneum. The rapture of primary hepatic and splenic cysts (either spontaneous or traumatic and iatrogenic) results in the formation of intraperitoneal hydatid cyst. 2 % of all the abdominal cases of hydatid cysts are consisted from primary intraperitoneal cyst which are very rare. Balik et al. had a retrospective review of 27 surgically treated extra hepatic cysts which took place between 1982 and 1999. Among them 19 patient had coexistent hepatic cysts (70.4 %) eight had only extra hepatic cysts (20.6 %) affecting the spleen (three patients), pancreas (two patients), adrenal glands (four patients), mesocolon (five patients), mesentery of the small intestine (one patient), ovaries (one patient), retroperitonem (four patients) and the omentum (two patients). When the hydatid embryo reaches to the mesentery by means of blood or lymph, it forms a solitary primary mesenteric cyst and no other cysts are present in this case. Mesenteric hydatid cyst can remain asymptomatic for many years and it becomes apparent when it grows causing mass effect on adjacent organs and making slowly growing mass in the abdomen [1]. Mass effect, peritoneal seeding rapture of the cyst, infection of the cyst, trans diaphragmatic involvement of the lungs, mediastinum and cardia are the complications of mesenteric hydatid cyst [6]. Hydatidosis can be by diagnosed by considering clinical findings, serologic tests accompanied by imaging studies such as plain radiography, ultrasonography, CT-scan, and MRI. The final definitive and preoperative diagnosis can be confirmed by radio-imaging studies (abdominal ultrasonography with computerized tomography) [6], [7].

The rarity of cases, lack of specific symptoms, and different appearance of imaging and quite resemblance to the organ's malignancy makes difficult reaching to a correct preoperative diagnosis. In differential diagnosis we should take in to account all the abdominal cystic lesions such us mesenteric, pancreatic, gastrointestinal duplication, ovarian cyst and lymphangia's [8].

Careful and complete surgical excision is the optimal treatment but sometimes in order to save other organs from being injured we can perform subtotal or partial cystectomy. To prevent from further spreading, anaphylaxis and to kill the daughter cyst, hypertonic saline or hydrogen peroxide can be used before opening the cavities. To prevent the recurrence Mebendazole and the Albendazole can be used as adjuvant therapy. In the cases in which there are disease recurrence or multiple sites are involved routinely usage of chemotherapy is preferred [9], [10].

The preoperative diagnosis has significant importance in preventing the spread of the cysts and seeding of the surgical field. Deaths have also been reported commonly as a result of anaphylactic shock which not only is caused from spillage during surgery but also by taking biopsy mistakenly diagnosed as an intraperitonem tumor [5]. Considering the possibly of hydatid cyst especially in endemic regions is highly recommended because in these regions there are diversity in the presentation. Radiography, ultrasonography, computed tomography, MRI, and immunological tests have significant value in diagnosing hydatid cysts [7].

## 4 Conclusion

Primary hydatid cyst of mesentery is rare even in the endemic regions. Thus, it's important to differentiate hydatid disease of abdomen from the other cystic lesions, occured in the abdominal cavity, specially in the endemic regions. Surgery is the treatment of choice however, it should be done with cautions not to spread the daughter cysts throughout the peritoneum and not to cause anaphylactic shock due to dissemination. In all cases of cystic masses in peritoneal cavity hydatid cysts should be considered in differential diagnosis.

## Provenance and peer review

Not commissioned; externally peer-reviewed.

## Funding

No financial support was provided for this study.

## Ethical approval

The manuscript has got an ethical review exemption from the Ethical Review Committee (ERC) of our institution, as case reports are exempted from review according to the institutional ethical review committee's policy.

## Consent

Written informed consent was obtained from the patient for publication of this case report and accompanying images. A copy of the written consent is available for review by the Editor-in-Chief of this journal on request.

## Author contribution

MAA convinced the idea. MAA and SHK operated the patient. MAA and RS was involved in literature review and drafted the manuscript. RS and MAA helped to collect clinical and follow-up data of the cases. RS and KW participated in reviewing the drafted manuscript. MAA participated with the corresponding, editing the drafted manuscript as per journal policy, and submission of the article. All authors read and approved the final manuscript.

## Registration of research studies

Not applicable.

## Guarantor

The corresponding author is the guarantor of article.

## Declaration of competing interest

The authors declare that their work is not funded by any institution, organ, or government and they have no financial support.
